# Application of Deep Eutectic Solvents in Food Analysis: A Review

**DOI:** 10.3390/molecules24244594

**Published:** 2019-12-16

**Authors:** Jingnan Chen, Yun Li, Xiaoping Wang, Wei Liu

**Affiliations:** 1 College of Food Science and Technology, Henan University of Technology, Lianhua Street 100, Zhengzhou 450001, China; chenjingnan813@hotmail.com (J.C.); 18660525535@163.com (X.W.); 2 Key Laboratory of Agro-products Safety & Quality of the Ministry of Agriculture, Institute of Quality Standards & Testing Technology for Agro-products, Chinese Academy of Agricultural Sciences, No.12, Zhongguancun South Street, Beijing 100081, China; liyun01@caas.cn

**Keywords:** deep eutectic solvents, food analysis, extraction, review

## Abstract

Deep eutectic solvents (DESs) have emerged recently as new and green solvents. DESs can be used for extraction and separation of both inorganic metallic components and organic compounds such as phenolic compounds, flavonoids, sugars, and aromatic amines from food samples. DESs possess a tunable property simply by adjusting the ratio of hydrogen bond acceptors to the hydrogen bond donors. As a green extraction medium, DESs have various applications in the pretreatment process and improve the efficiency of different food analyses. This review summarizes the findings of recent studies on the development, production, application, and efficacy of DESs in the pretreatment process of various food analyses.

## 1. Introduction

Food analysis has become one of the most important procedures in safety and quality control during the manufacture of food products. Many novel analytical methods, including sample pretreatment and instrumental techniques, have been developed. On the one hand, it is essential to develop various new instrumental techniques regarding the applications of ultra-performance liquid chromatography (UPLC) and liquid chromatography coupled to triple quadrupole mass spectrometry in food analysis. On the other hand, it is equally important to develop various novel pretreatment technologies with low cost and high efficiency in analysis of foods.

Conventional organic solvents, such as methanol, acetonitrile, ethanol, hexane, acetones, and so on, are required in pretreatment technologies for food analysis, including extraction, separation, and pre-concentration. These conventional organic solvents are of low cost and are easy to evaporate; however, their residue levels and resulting pollution are of concern [1]. With the demand for sustainable and green processes, the development of green solvents for food analysis has therefore received much attention in the past ten years [2,3,4].

With the aim of developing environmentally friendly solvents, deep eutectic solvents (DESs) have emerged recently as new and green solvents [5,6]. DESs can be easily synthesized by mixing a hydrogen bond acceptor (HBA) and a hydrogen bond donor (HBD) after mild heating (60–90 °C) for 30 min to one hour [7]. The most common HBAs used are cheap quaternary ammonium salts (e.g., choline chloride), while HBDs are always urea, ethylene glycol, glycerol, amino acids, carboxylic acids, and sugars. Because most of the starting materials for DESs exist naturally, DESs present the properties of having high purity and being environmental friendly [7,8]. DESs have been widely applied in the extraction, separation, and pre-concentration of target components, including phenolic compounds [9], flavonoids [10], sugars [11], proteins, metals, and so on.

The viscosity of solvents affects the extraction efficiency. In this regard, the viscosity of DESs is generally higher than that of conventional organic solvents. The viscosity of the DESs can be adjusted by changing the concentrations and mole ratio of HBAs and HBDs as well as temperature during the extraction. Liu et al. found that the viscosity of a DES composed of ascorbic acid and choline chloride (ChCl) decreased rapidly from the original 3.799 mPa·s to 0.1203 mPa·s with the addition of 10% water (v/v) [12]. The viscosity of the DES (ascorbic acid/ChCl/water) also decreased by 80% when the temperature was elevated from 30 °C to 70 °C. In addition, the polarity of DESs, which is very close to that of most organic polar solvents, is related to their components and the addition of a small portion of water. In the absence of water, the polarity of DESs synthesized by different components is in the order of organic acid-based DESs > amino acid-based DESs > sugar-based DESs > polyol-based DESs [3,5].

**Figure 1:**
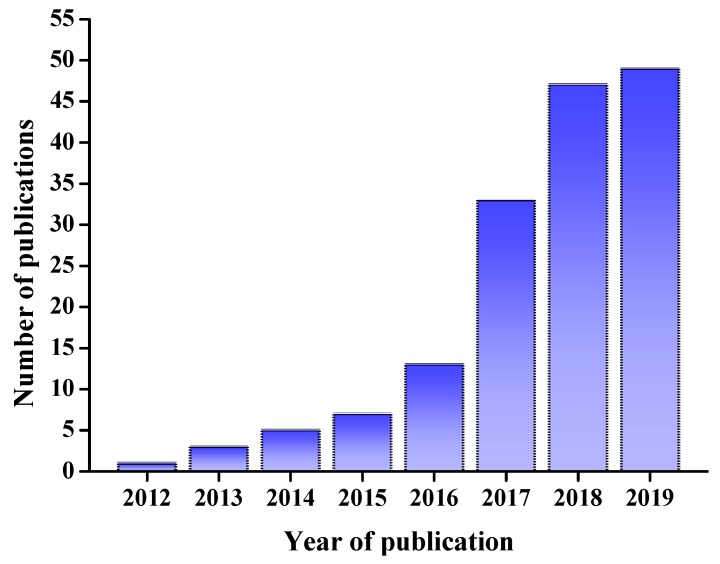
Publications using “DES” as a key word in recent years from the Web of Science (WoS). DES: deep eutectic solvent.

DESs have numerous applications in the analyses of minor components in foods. It is worth noting that the number of recent publications regarding the applications of DESs in the field of food science and technology in the Web of Science (WoS) has increased significantly, with about 50 publications each year in 2018 and 2019 (Figure 1). The present review aims to summarize recent research and our own work on applications of DESs in food analyses.

## 2. DESs for the Extraction of Phenolic Compounds

### 2.1. DESs for the Extraction of Synthetic Phenolics

Phenolic compounds are used as antioxidants to delay the rancidity of edible oils. Tert-Butylhydroquinone (TBHQ) is one of the most commonly used synthetic phenolic antioxidants in edible oils (e.g., soybean oil and blend oil) ascribed to its low cost. A maximum limit of 0.02% TBHQ is permitted in foods in many countries, such as China, the United States, and Australia. Thus, accurate determination of TBHQ is very important for food safety. An HPLC method based on traditional liquid–liquid extraction (LLE) has been widely used for the analysis of TBHQ. In 2017, Liu et al. developed a green and inexpensive ultrasonic-assisted liquid–liquid micro-extraction (UALLME) technique based on DESs for the extraction of TBHQ from edible oils, which could be combined with HPLC detection [13]. The results indicated that the DESs made from ChCl and ethylene glycol (1:2 M ratio) showed a higher extraction efficiency of TBHQ (close to 100%) than ChCl/propylene glycol (1:2 M ratio) and ChCl/glycerol (1:2 M ratio). Subsequently, a novel natural deep eutectic solvent (NADES) synthesized by ChCl and ascorbic acid was also used for UALLME of TBHQ from edible oils [12], coupled with the HPLC-UV analysis. The results showed that the NADES consisting of ChCl and ascorbic acid (2:1 M ratio) exhibited a higher extraction efficiency (close to 100%) and excellent protection ability for TBHQ during the extraction process. Similarly, extraction and pre-concentration of synthetic phenolic antioxidants alkyl gallates from vegetable oils were achieved by hydrophilic DESs [14]. In this work, a DES composed of ChCl and ethylene glycol (1:2 M ratio) was adopted for LLE from vegetable oil samples, and then the DES phase (containing alkyl gallates) was detected by LC-UV.

### 2.2. DESs for the Extraction of Natural Phenolics

Plant-derived foods contain numerous kinds of phenolic compounds, which are mainly affected by the cultivated varieties, food processing, and food preservation. As these phenolic compounds possess various biologically activities and are nutritionally beneficial to humans, the analysis of phenolic compounds is of importance for the food industry.

**Figure 2:**
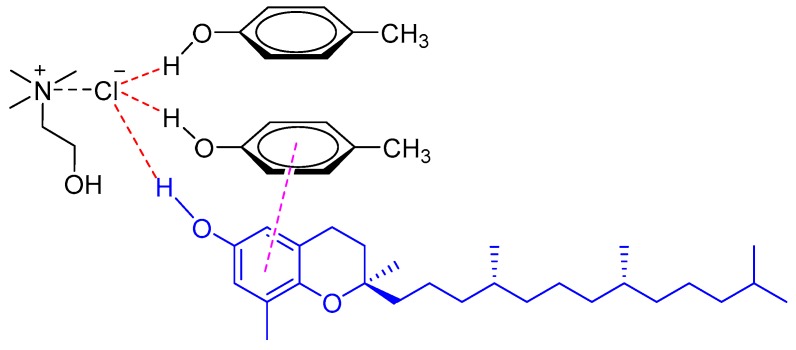
The interaction of a DES (composed of ChCl and *p*-cresol) with tocopherol through π−π interaction.

Natural phenolic compounds in edible oils, such as tocols [15,16] and sesamol [17], could be rapidly extracted or pre-concentrated with various DESs. Hadi et al. investigated the performance of choline-based DESs in the extraction of tocopherols and tocotrienols (collectively known as tocols) from crude palm oil [15]. The results showed that the DESs comprised of ChCl and carboxylic acids were effective for the extraction of tocols from crude palm oil by LLE. The tocol concentration in the DES extracts reached up to 18,525 mg/kg with increasing amounts of the DES. Recently, a vortex-assisted liquid–liquid extraction (VALLE) method based on phenolic DES was developed for extraction of tocopherols from soybean oil deodorizer distillate (SODD), the main natural source of tocopherols (V*_E_*) [16]. It was found that phenolic DES made from ChCl and *p*-cresol (1:2 M ratio) showed the best extraction efficiency at room temperature for all tocopherols isomers (alpha, gamma, and delta-tocopherols) due to the strong π−π interaction between the phenolic DES and tocopherols (Figure 2).

Sesamol is an important natural antioxidant in sesame oils. In 2017, Liu et al. developed a simple UALLME technique based on choline-based DESs for the extraction of sesamol from sesame oils [17]. It was found that the DES composed of ChCl and ethylene glycol (1:2 M ratio) was efficient for micro-extraction of sesamol from various sesame oil samples, and the detected results were close to those of conventional LLE with methanol as a solvent.

UALLME method based on DESs could be used for the extraction of three phenolic acids (ferulic, caffeic, and cinnamic) from olive, almond, sesame, and cinnamon oil [18]. The study demonstrated that DESs prepared from ChCl combined with ethylene glycol and/or glycerol were better than ethylene glycol or glycerol alone for extraction and pre-concentration of target analytes at a trace level. In succession, a similar work for extraction of phenolic compounds from virgin olive oil was also reported [19]. The authors evaluated the extraction efficiency of a set of DESs consisting of ChCl in various mixing ratios with sugars, alcohols, organic acids, and urea for the two most abundant secoiridoid derivatives in olive oil (oleacein and oleocanthal), finding that ChCl/xylitol and ChCl/1,2-propanediol could increase the extraction efficiency by 30–70% compared with conventional methanol/water extraction.

Moreover, DESs based on natural chemicals (glucose and lactic acid) were used for extraction of phenolic compounds from olive oils. Paradiso et al. found that addition of a small amount of water into a DES based on glucose and lactic acid (1:6 M ratio) could reduce the viscosity of solvent [20] Similarly, a NADES composed of glucose, lactic acid, and water (3:1:3 M ratio) was used to extract hydroxytyrosol and tyrosol derivatives from olive oils, a health claim confirmed by the European Regulations for olive oils [21]. After extraction with NADES, phenolic compounds were then spectrophotometrically quantified directly.

DESs have been also applied to polyphenols from grapefruit peels [22]. In order to increase the extraction efficiency, a high-voltage electrical discharge was used as a pre-treatment technology. The subsequent liquid–solid extraction (LSE) was performed in DES (lactic acid/glucose) at a molar ratio of 5:1. Interestingly, the addition of glycerol in extraction could reduce the energy of the pre-treatment by six times.

**Figure 3:**
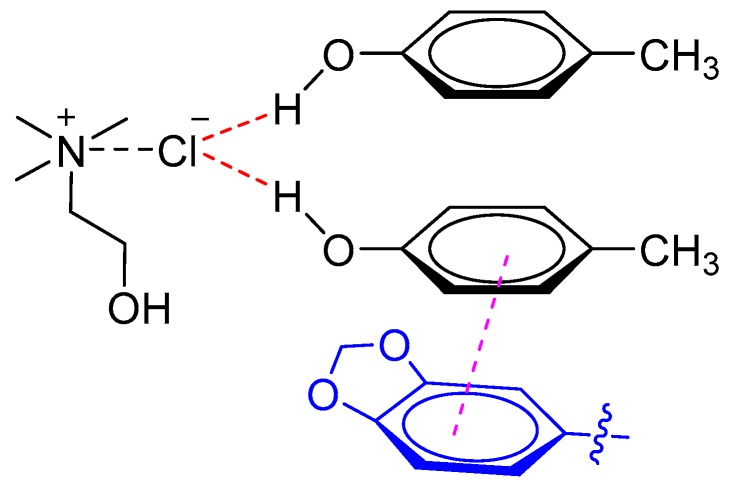
Phenolic DES interaction with sesamin or sesamolin through π–π stacking.

Recently, Liu et al. established a highly efficient UALLME technique based on DESs for determination of lignans in sesame oils [23]. The results demonstrated that DESs composed of ChCl and *p*-cresol (1:2 M ratio) had a better ability for simultaneous extraction of three lignans (i.e., sesamin, sesamolin, and sesamol) than common polyols-based DESs composed of polyols (e.g., ethylene glycol and glycerol) and ChCl. It was proposed that the phenolic DESs (ChCl and *p*-cresol) exhibited higher extraction efficiency for both polar lignans (i.e., sesamol) and non-polar lignans (i.e., sesamin and sesamolin) due to the π–π interaction between lignans and DESs (Figure 3).

A DES as a green solvent coupled with highly efficient microwave-assisted extraction (MAE) and ultrasound-assisted extraction methods (UAE) was developed to extract phenolic compounds from grape skin [24]. On screening of different DESs (ChCl/glycerol, ChCl/oxalic acid, ChCl/malic acid, and ChCl/sorbose), it was found that the ChCl-based DES containing oxalic acid as a HBD with 25% (v/v) of water was more effective in extracting phenolic compounds from grape skin compared to conventional solvents (e.g., water, aqueous methanol, and acidified aqueous solution of methanol). Additionally, to obtain environmentally friendly polyphenolic extracts from grape and olive pomace, NADESs coupled with ultrasound and microwave irradiation were developed [25]. In this work, DESs comprised of ChCl and citric acid (2:1 M ratio) could be simply prepared by mixing at 50 °C for 2 h and then diluting with 30% (v/v) of water. The resultant polyphenolic extracts in the NADES (ChCl/citric acid) could be considered as ready-to-use in food industry without going through expensive purification steps.

Curcumin, the major component of curcuminoids, is an important polyphenolic pigment. Vortex assisted DES-emulsification liquid–liquid micro-extraction was developed for separation and pre-concentration of trace curcumin in food and herbal tea samples [26]. A DES consisting of ChCl and phenol (1:4 M ratio) was prepared to extract curcumin at pH 4.0 with tetrahydrofuran (THF) as an emulsifier agent in micro-extraction. The curcumin concentration in enriched DES phase could be analyzed by UV-Visible spectrophotometer at 425 nm (λ*max*).

Ferrone et al. evaluated the DESs based on carboxy-betaine, including glycolic acid/trimethylglicine, phenylacetic acid/trimethylglicine, 2-furoic acid/trimethylglicine, and mandelic acid/trimethylglicine on the simultaneous quantification of ferulic acid, umbelliferone, boropinic acid, 7-isopentenyloxycoumarin, 4′-geranyloxyferulic acid, and auraptene in some vegetable oils (e.g., olive, soy, peanuts, corn, and sunflower oil) coupled with UPLC with photodiode array detection [27]. It was demonstrated that the DES composed of phenylacetic acid and trimethylglicine was the most promising solvent for the quantification of the six target compounds simultaneously.

Peppermint (*Mentha piperita* L.) is a very popular non-caffeinated herbal tea due to its beneficial effects, such as the antioxidant and antimicrobial properties ascribed to the contents of volatile monoterpenes and phenolic compounds. DESs that could be used for one-step preparation for chemical characterization of peppermint were prepared by Jeong et al. [28], who found that the DES composed of ChCl and D-(+)-glucose (5:2 M ratio) was the most efficient extraction solvent of the selected DESs (HBA: citric acid, urea, or ChCl; HBD: glycerol, xylitol, or D-(+)-glucose).

## 3. DESs for the Extraction of Flavonoids

NADES is a promising green solvent for the extraction of flavonoids from natural and argo-products [29,30]. A green sample preparation method based on NADES was developed for the extraction of a series of flavonoids (rutin, hesperidin, neohesperidin, naringenin, naringin, quercetin, hesperetin, and chrysin) from fruits, vegetables, and spices [31]. The extraction efficiency of NADES based on ChCl, acetylcholine chloride, choline tartrate, betaine, and carnitine with different compositions were evaluated, and it was found that NADES composed of acetylcholine chloride and lactic acid (2:1 molar ratio) diluted with 30% water had a highest recovery of flavonoids (higher than 70%).

Mansur et al. investigated the extraction efficiency of 18 different ChCl-based DESs on the flavonoids from common buckwheat sprouts coupled with the UAE method [32]. It was found that an 80% ChCl-based DES (ChCl/triethylene glycol) with 20% (v/v) water extracted significantly higher amounts of flavonoids than other DESs, even higher than that of methanol for the extraction of vitexin and quercetin-3-O-robinobioside. Most importantly, flavonoids could be efficiently recovered from DES extracts with high recovery yields (>97%) by using a C18 solid-phase extraction.

The citrus-processing industry generates a large amount of citrus peel waste as by-product, accounting for nearly 50% of the wet fruit mass. The citrus peels are a rich source of flavonoids and pectin. Using ChCl-based DESs as green alternatives, valuable flavonoids could be obtained through extracting under ultrasonic irradiation conditions [33]. It was found that a ChCl-levulinic acid-N-methyl urea DES showed a highest extraction yield of total flavonoids (polymethoxylated flavonoids and glycosides of flavonoids) from citrus peel.

Guo et al. utilized NADES coupled with high-speed homogenization and cavitation-burst extraction to extract anthocyanins from fresh mulberry [34]. The results implied that the NADES with organic acids as the HBDs had a better extraction efficiency for anthocyanin extraction than those with sugars or sugar alcohols. The NADES comprised of ChCl-citric acid-glucose with the mole ratio of 1:1:1 (containing 30% water) showed the best extraction efficiency of anthocyanins as compared to traditional organic solvents. Similarity, Panic et al. obtained anthocyanins from grape pomace successfully using NADES extraction [35]. The NADES composed of ChCl and citric acid (2:1 M ratio) with 30% (v/v) of water presented the best extraction efficiency under U/MAE conditions. Importantly, this developed procedure could be scaled up to a half-liter batch, which provided a potential application in large-scale production in food industry.

## 4. DESs for the Extraction of Other Polar Organic Compounds

Food contaminants, including synthetic dye, herbicides and toxins, are raising concerns for food safety nowadays. The effect of DESs on the liquid-phase micro-extraction (LPME) of plant growth regulators, including indole-3-acetic acid, indole-3-butyric acid, and 4-iodophenoxyacetic acid, from various edible vegetable oil samples was examined by Tan et al. [36]. The results demonstrated that a DES composed of tetramethylammonium chloride-ethylene glycol (1:3 M ratio) exhibited the best extraction efficiency for the three plant growth regulators from edible oil samples.

Rhodamine B is used as an artificial synthetic dye, and its use is forbidden in the food industry. However, rhodamine B is often added illegally in food due to its low cost, especially in chili oils. Yang et al. established a DES extraction method coupled with UPLC analysis for the determination of trace rhodamine B in chili oil [37], demonstrating that the DES comprised of ChCl and ethylene glycol (1:3 M ratio) was efficient for selective extraction of rhodamine B from chili oils.

**Figure 4:**
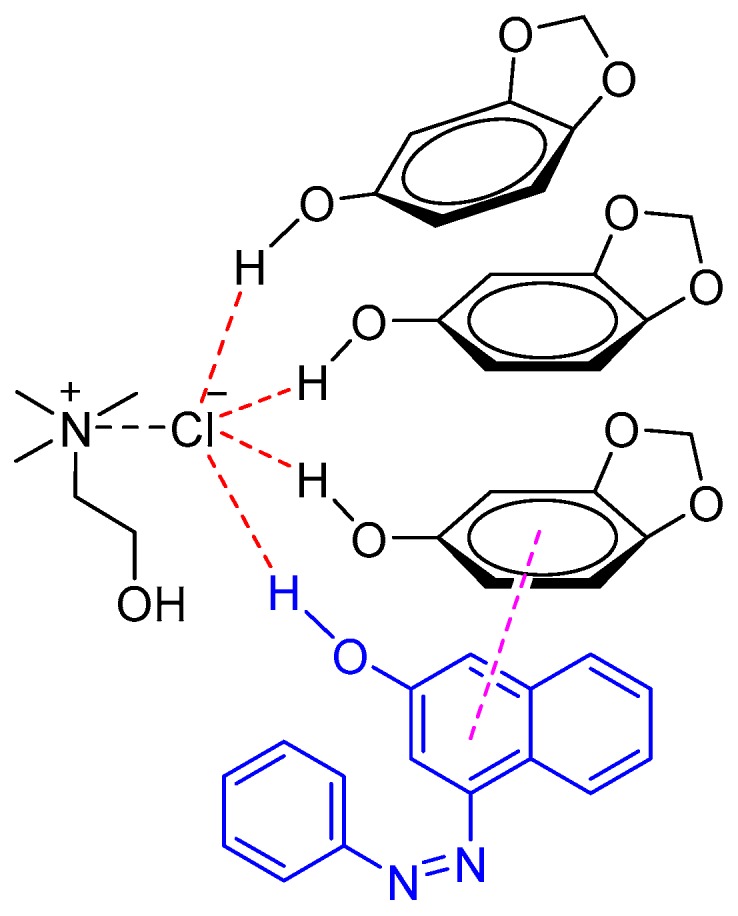
Natural deep eutectic solvent (NADES) (sesamol/ChCl) interaction with Sudan I through π–π stacking and the hydrogen bond.

Sudan I is the most widely used synthetic lipophilic azo dye in the chemical industry. In the food industry its use is absolutely forbidden. Recently, Liu et al. developed a NADES system to identify Sudan I in food samples such as chili oil, chili sauce, and duck egg yolk [38]. It was found that the NADES composed of sesamol and ChCl (3:1 M ratio) exhibited better extraction abilities of Sudan I as compared to other tested DESs (e.g., phenol/ChCl, ethylene glycol/ChCl, and glycerol/ChCl) and conventional organic solvents (e.g., methanol). The excellent extraction abilities were attributed to the π–π and hydrogen bond interaction between NADES (sesamol/ChCl) and Sudan I (Figure 4).

Pharmaceuticals and personal care products (PPCPs), as emerging pollutants, persistently enter the aqueous environment and accumulate in aquatic organisms. Liu et al. investigated the DES-based LPME for the determination of pharmaceuticals and personal care products in fish oil, and the extraction of four common PPCPs (sulfamethazine, sulfamethoxazole, triclocarban, and carbamazepine) in fish oil was achieved using DESs [39]. Wang et al. evaluated DESs-based liquid–liquid micro-extraction (LLME) on the four triazine herbicides (namely, simazine, ametryn, prometryn, and terbuthylazine) from edible oil samples [40], finding that the DES formed by tetrabutylammonium chloride ((N4444)Cl, TBA) and ethylene glycol was crucial for the vortex-assisted reverse-phase liquid–liquid micro-extraction.

Aflatoxins (AFs) are highly toxic and carcinogenic mycotoxins produced by the secondary metabolism of certain *Aspergillus* species. Considering their threat to food safety, sensitive and accurate determination of AFs is currently an important requirement to meet official regulations. A novel and sensitive DES-based matrix solid-phase dispersion method for the determination of aflatoxins (AFB1, AFB2, AFG1, AFG2) in various crops (such as millet, peanut, and heepseed) was established using HPLC with fluorescence detection [41]. Considering the solubility of AFs, three types of TBAC-based DESs (TBAC-hexyl alcohol, TBAC-dodecyl alcohol, and TBAC-hexanoic acid) were selected as extraction mediums, and it was found that the DES composed of TBAC and hexyl alcohol showed the best recoveries (93.67 ± 3.28%~98.07 ± 1.10%) for all AFs (AFB1, AFB2, AFG1, AFG2).

Drinking water is easily contaminated by hazardous chemical compounds (e.g., aromatic amines). An efficient and rapid method for trace analysis of aromatic amines in water samples was developed based on DESs [42]. In this work, air-assisted LLME based on solidification of lighter-than-water DES coupled with GC-MS was used for detecting aromatic amines, including aniline, *p*-toluidine, *p*-chloroaniline, *p*-anisidine and 4-*tert*-butyl aniline.

Ethanolic extracts of propolis are consumed for their health benefits. Owing to the biocompatibility, biodegradability, and renewability of NADESs, they were used to extract propolis to substitute alcohol [43]. Sixteen NADESs prepared from non-toxic materials (e.g., essential amino acids) were successfully used for the green extraction of propolis including its biomarker artepillin C. Most importantly, the extracts of propolis based on NADES were much more stable over the months and could be consumed directly with no need for the separation of the bioactive compounds from NADES. In recent years, with the aim of rapid separation from samples, magnetic DESs comprised of inexpensive components [ChCl/*para*-cresol] [FeCl_4_] were synthesized and utilized successfully in UALLME and back-extraction methods to identify hexanal and heptanal in edible oils [44]. The merit of this novel DES is that it is able to be magnetic recycled easily.

## 5. DESs for the Extraction and Pre-Concentration of Metals

Food samples (e.g., edible oils) may be contaminated by trace amounts of metals originating from different sources (e.g., soil and the environment) or introduced during storage and refining processes. The quality and safety of food samples is directly related to the concentration of trace metals, especially the toxic metals. Thus, the determination of trace metals in various food samples is significantly important from the health point of view. In this regard, DESs composed of ChCl and urea (1:2 M ratio) were used in LPME combined with electro-thermal atomic absorption spectrometry (ETAAS) for separation, pre-concentration, and determination of lead (Pb) and cadmium (Cd) in various edible oils [45]. Moreover, Cd could be efficiently removed from rice flour using NADES washing [46]. ChCl-based NADESs, including ChCl/glycerol/water, ChCl/tartaric acid/water, ChCl/xylitol/water, and ChCl/malic acid/water, all exhibited good Cd removal ability (51–96%) from rice flour. Similarly, Zounr et al. developed a simple and fast ultrasonic assisted DES-LPME technique for the pre-concentration and extraction of Cd in water and food samples by ETAAS [47], demonstrating that the DES formed by ChCl and phenol (1:4 M ratio) had the maximum extraction efficiency for Cd (>95%). DES-LPME for the determination of Pb at trace levels was achieved by slotted quartz tube-flame atomic absorption spectrometry [48]. The DES comprised of ChCl and phenol (1:2 molar ratio) showed a maximum extraction efficiency for Pb from spiked raw milk samples.

Considering the negative impact on human health, determination of arsenic (As) and antimony (Sb) is also necessary for food safety. Recently, Altunay et al. reported a practical DES based vortex assisted micro-extraction (VAME) method for pre-concentration of As and Sb from honey and rice samples prior to analysis by hydride generation atomic absorption spectrometry [49], finding that the DES prepared by ChCl and oxalic acid (1:4 M ratio) performed the best extraction recovery for the pre-concentration of As and Sb simultaneously.

## 6. DESs for the Extraction Protein

DESs can be also applied in protein extraction. Liu et al. investigated the extraction of pumpkin seed protein with poly (ethylene glycol)-based DES under ultrasound-microwave synergistic extraction conditions [50]. It was found that aqueous poly (ethylene glycol) (PEG 200)-based DES (ChCl/PEG200 1:3 M ratio) possessed the highest extraction efficiency. Furthermore, an isoelectric point/ethanol/PEG 200-based DES co-precipitation method was successfully applied to the precipitation of pumpkin seed protein from the extraction solution.

Gluten analysis is a very important issue in the food industry. An effective gluten extraction method was established using ChCl-based DESs [51]. In this investigation, the ChCl based DESs using ethylene glycol or urea as a HBD were selected as extraction media from both unprocessed and processed food. Importantly, a high recovery was found using these DESs comparison with 60% (v/v) ethanol–water solvent. Moreover, enhanced protein extraction from oilseed cakes (e.g., rapeseed) was developed using DES composed of glycerol-ChCl [52]. The protein-rich precipitates could be obtained by adding water as anti-solvent to the DES (glycerol-ChCl 2:1 M ratio) extract derived at 60 °C.

## 7. Hydrophobic DESs for the Extraction of Food Samples

A typical DES is made from a quaternary ammonium salt (e.g., ChCl) and an HBD (e.g., urea, glycerol, sugar, or carboxylic acid) to provide a hydrophilic DES. This type of DESs is water-soluble, limiting its application to water-containing samples. Therefore, the development of hydrophobic DESs is highly desirable [53]. Faraji et al. utilized a hydrophobic DES composed of tetrabutylammonium chloride and octanoic acid (1:2 M ratio) as a micro-extraction solvent coupled with HPLC analysis to extract and determine synthetic pigments, including lemon yellow, amaranth, sunset yellow, indigo, carmine, allura red, brilliant blue, and erythrosine [54]. Effervescence-assisted dispersive liquid–liquid micro-extraction (DLLME) based on a hydrophobic DES has been developed to extract synthetic dyes (Sunset yellow and Brilliant blue FCF) from food samples [55]. The hydrophobic DES consisting of aliquat 336 and oleic acid (1:2 M ratio) exhibited the most efficient extraction ability for aqueous food samples.

Similarity, Faraji et al. reported vortex-assisted DES-DLLME of five commonly used synthetic red dyes (amaranth, ponceau 4R, allura red, azorubine, and erythrosine) in food samples and simultaneous determination by HPLC [56]. In this work, a novel hydrophobic DES made from thymol and benzyltriethylammoniumchloride, could be prepared at 70 °C within only 15 min. This simple and sensitive method was successfully applied in analyzing the food colorants in beverage, jelly, and chocolate samples with satisfactory results (recoveries ranged from 94.2% to 100.8%).

Pyrethroids are increasingly used in modern agriculture for pest control, and pyrethroid residues have been widely found in agricultural foods. Using hydrophobic DESs as extraction solvents, the DLLME method was established for extraction of pyrethroids in real tea beverages and fruit juices [57]. In the related study, a series of new hydrophobic DESs were prepared using hexafluoroisopropanol as an HBD and L-carnitine/betaine as an HBA. The proposed DLLME method coupled with HPLC showed good performance for extraction and determination of pyrethroids (transfluthrin, fenpropathrin, fenvalerate, ethofenprox, and bifenthrin) in food samples.

## 8. General Considerations Using DESs as Extraction Solvents

In the extraction or separation of organic or inorganic targets from food samples, DESs were selected according to the states of the food samples, including liquid food (e.g., edible oils, tea beverage, carbonated drinks, fruit juices, and water) and solid food (e.g., corn, rice flour, and oilseed cakes). Therefore, DESs combined with LLE or LLME are suitable for the extraction of liquid food samples, while DESs with LSE are suitable for solid food samples. DESs can be classified into hydrophilic and hydrophobic types. As an extraction medium, the polarity of DESs should be considered firstly. Summarizing the findings, hydrophobic DESs are suitable for the extraction of organic and inorganic analytes from aqueous food samples through LLE or LLME methods, while hydrophilic DESs are adequate for the extraction of analytes from low-polar food samples, such as edible oils. In most cases, ultrasound-assisted micro-extraction, ultrasound-assisted micro-extraction, and microwave-assisted micro-extraction are commonly chosen due to their extraction efficiency. As DESs have a low volatility and are non-flammable, the micro-extraction based on DES extracts can be coupled with HPLC or other detection methods (e.g., UV-Vis) for the determination of targets without further reverse extraction. Generally speaking, UV-Vis can be used for detecting a single analyte in DES extracts. DES extracts containing more than one analyte would be determined with HPLC or GC methods.

Most importantly, DESs can be used as sustainable solvents from gram-scale to liter-scale [35] due to their low cost of starting materials (urea, ChCl, sugars, and organic acids). Though gram-scale use of DES in LLE or LSE is appropriate for food analysis process, scale-up use of DES will be highly desirable for various food and by-product process. DESs composed of ChCl and glycerol were successfully used for protein extraction from oilseed (i.e., rapeseed) cake [52]. In this work, 45 g DES were utilized for treatment of a 5 g sample (oilseed cake) and the reuse of DES was also conducted, indicating the potential large-scale applications of DESs.

## 9. Conclusions

DESs as new and green solvents have been used for food analysis through pretreatments (e.g., extraction or separation) of food samples. Hydrophilic DESs or hydrophobic DESs were selected according to the properties of the extraction targets and food samples (liquid or solid foods). The properties of DESs can be adjusted through mixing the HBAs and HBDs at different molar ratios. Moreover, water is commonly added to reduce the viscosity of DESs for rapid mass transfer between the target and extraction solvents. Compared with conventional organic solvents, hydrophilic DESs and hydrophobic DESs are non-volatile, so they can be combined with HPLC and UV-Visible spectrophotometry for rapid analysis. In fact, switchable DESs coupled with different micro-extraction technologies, including LLME and liquid–solid micro-extraction, are the main focus in the development of food analysis. Furthermore, NADESs are also highly desirable due to their natural and non-toxic essence, which will provide a sustainable alternative for the food industry.

## References

[1] Puranik S.B., Sanjay Pai P.N., Rao G.K. (2009). Determination of organic volatile impurities in herbal formulations and extracts by capillary gas chromatography. Pharmacogn. Mag..

[2] Murador D.C., Mesquita L.M.D., Vannuchi N., Braga A.R.C., de Rosso V.V. (2019). Bioavailability and biological effects of bioactive compounds extracted with natural deep eutectic solvents and ionic liquids: Advantages over conventional organic solvents. Curr. Opin. Food. Sci..

[3] Choi Y.H., Verpoorte R. (2019). Green solvents for the extraction of bioactive compounds from natural products using ionic liquids and deep eutectic solvents. Curr. Opin. Food. Sci..

[4] Cunha S.C., Fernandes J.O. (2018). Extraction techniques with deep eutectic solvents. Trac-Trend. Anal. Chem..

[5] Smith E.L., Abbott A.P., Ryder K.S. (2014). Deep Eutectic Solvents (DESs) and their Applications. Chem. Rev..

[6] Zhang Q.H., Vigier K.D., Royer S., Jerome F. (2012). Deep eutectic solvents: Syntheses, properties and applications. Chem. Soc. Rev..

[7] Abbott A.P., Boothby D., Capper G., Davies D.L., Rasheed R.K. (2004). Deep eutectic solvents formed between choline chloride and carboxylic acids: Versatile alternatives to ionic liquids. J. Am. Chem. Soc..

[8] Rub C., Konig B. (2012). Low melting mixtures in organic synthesis-an alternative to ionic liquids?. Green Chem..

[9] Ruesgas-Ramon M., Figueroa-Espinoza M.C., Durand E. (2017). Application of deep eutectic solvents (DES) for phenolic compounds extraction: Overview, challenges, and opportunities. J. Agric. Food Chem..

[10] Nam M.W., Zhao J., Lee M.S., Jeong J.H., Lee J. (2015). Enhanced extraction of bioactive natural products using tailor-made deep eutectic solvents: Application to flavonoid extraction from Flossophorae. Green Chem..

[11] Das A.K., Sharma M., Mondal D., Prasad K. (2016). Deep eutectic solvents as efficient solvent system for the extraction of k-carrageenan from Kappaphycusalvarezii. Carbohydr. Polym..

[12] Liu W., Zhang K.D., Chen J.N., Yu J.J. (2018). Ascorbic acid and choline chloride: A new natural deep eutectic solvent for extracting tert-butylhydroquinone antioxidant. J. Mol. Liq..

[13] Liu W., Zhang K.D., Yu J.J., Bi Y.L. (2017). A green ultrasonic-assisted liquid-liquid microextraction based on deep eutectic solvent for the HPLC-UV determination of TBHQ in edible oils. Food Anal. Methods.

[14] Çabuk H., Yılmaz Y., Yıldız E. (2019). A vortex-assisted deep eutectic solvent-based liquid-liquid microextraction for the analysis of alkyl ggallates in vegetable oils. Acta Chim. Slov..

[15] Hadi N.A., Ng M.H., Choo Y.M., Hashim M.A., Jayakumar N.S. (2015). Performance of choline-based deep eutectic solvents in the extraction of tocols from crude palm oil. J. Am. Oil Chem. Soc..

[16] Liu W., Fu X.L., Li Z.Z. (2019). Extraction of tocopherol from soybean oil deodorizer distillate by deep eutectic solvents. J. Oleo Sci..

[17] Liu W., Zhang K.D., Qin Y.Q., Yu J.J. (2017). A simple and green ultrasonic-assisted liquid liquid microextraction technique based on deep eutectic solvents for the HPLC analysis of sesamol in sesame oils. Anal. Methods.

[18] Khezeli T., Daneshfar A., Sahraei R. (2016). A green ultrasonic-assisted liquid-liquid microextraction based on deep eutectic solvent for the HPLC-UV determination of ferulic, caffeic and cinnamic acid from olive, almond, sesame and cinnamon oil. Talanta.

[19] Garcia A., Rodriguez-Juan E., Rodriguez-Gutierrez G., Rios J.J., Fernandez-Bolanos J. (2016). Extraction of phenolic compounds from virgin olive oil by deep eutectic solvents (DESs). Food Chem..

[20] Paradiso V.M., Clemente A., Summo C., Pasqualone A., Caponio F. (2016). Towards green analysis of virgin olive oil phenolic compounds: Extraction by a natural deep eutectic solvent and direct spectrophotometric detection. Food Chem..

[21] Paradiso V.M., Squeo G., Pasqualone A., Caponio F., Summo C. (2019). An easy and green tool for olive oils labelling according to the contents of hydroxytyrosol and tyrosol derivatives: Extraction with a natural deep eutectic solvent and direct spectrophotometric analysis. Food Chem..

[22] El Kantar S., Rajha H.N., Boussetta N., Vorobiev E., Maroun R.G., Louka N. (2019). Green extraction of polyphenols from grapefruit peels using high voltage electrical discharges, deep eutectic solvents and aqueous glycerol. Food Chem..

[23] Liu W., Zhang K.D., Yang G.L., Yu J.J. (2019). A highly efficient microextraction technique based on deep eutectic solvent formed by choline chloride and p-cresol for simultaneous determination of lignans in sesame oils. Food Chem..

[24] Bubalo M.C., Curko N., Tomasevic M., Ganic K.K., Redovnikovic I.R. (2016). Green extraction of grape skin phenolics by using deep eutectic solvents. Food Chem..

[25] Panic M., Stojkovic M.R., Kraljic K., Skevin D., Redovnikovic I.R., Srcek V.G., Radosevic K. (2019). Ready-to-use green polyphenolic extracts from food by-products. Food Chem..

[26] Aydin F., Yilmaz E., Soylak M. (2018). Vortex assisted deep eutectic solvent (DES)-emulsification liquid-liquid microextraction of trace curcumin in food and herbal tea samples. Food Chem..

[27] Ferrone V., Genovese S., Carlucci M., Tiecco M., Germani R., Preziuso F., Epifano F., Carlucci G., Taddeo V.A. (2018). A green deep eutectic solvent dispersive liquid-liquid micro-extraction (DES-DLLME) for the UHPLC-PDA determination of oxyprenylated phenylpropanoids in olive, soy, peanuts, corn, and sunflower oil. Food Chem..

[28] Jeong K.M., Jin Y., Yoo D.E., Han S.Y., Kim E.M., Lee J. (2018). One-step sample preparation for convenient examination of volatile monoterpenes and phenolic compounds in peppermint leaves using deep eutectic solvents. Food Chem..

[29] Liu Y., Friesen J.B., McAlpine J.B., Lankin D.C., Chen S.N., Pauli G.F. (2018). Natural deep eutectic solvents: Properties, applications, and perspectives. J. Nat. Prod..

[30] Paiva A., Craveiro R., Aroso I., Martins M., Reis R.L., Duarte A.R.C. (2014). Natural deep eutectic solvents—Solvents for the 21st Century. ACS Sustain. Chem. Eng..

[31] Bajkacz S., Adamek J. (2018). Development of a method based on natural deep eutectic solvents for extraction of flavonoids from food samples. Food Anal. Methods.

[32] Mansur A.R., Song N.E., Jang H.W., Lim T.G., Yoo M., Nam T.G. (2019). Optimizing the ultrasound-assisted deep eutectic solvent extraction of flavonoids in common buckwheat sprouts. Food Chem..

[33] Xu M.L., Ran L., Chen N., Fan X.W., Ren D.B., Yi L.Z. (2019). Polarity-dependent extraction of flavonoids from citrus peel waste using a tailor-made deep eutectic solvent. Food Chem..

[34] Guo N., Ping K., Jiang Y.W., Wang L.T., Niu L.J., Liu Z.M., Fu Y.J. (2019). Natural deep eutectic solvents couple with integrative extraction technique as an effective approach for mulberry anthocyanin extraction. Food Chem..

[35] Panic M., Gunjevic V., Cravotto G., Redovnikovic I.R. (2019). Enabling technologies for the extraction of grape-pomace anthocyanins using natural deep eutectic solvents in up-to-half-liter batches extraction of grape-pomace anthocyanins using NADES. Food Chem..

[36] Tan T., Li Z., Mao X.J., Wan Y.Q., Qiu H.D. (2016). Deep eutectic solvent-based liquid-phase microextraction for detection of plant growth regulators in edible vegetable oils. Anal. Methods.

[37] Wang W.D., Du Y.G., Xiao Z.E., Li Y., Li B.F., Yang G.W. (2017). Determination of trace rhodamine B in chili oil by deep eutectic solvent extraction and an ultra high-performance liquid chromatography equipped with a fluorescence detector. Anal. Sci..

[38] Liu W., Zong B.Y., Wang X.P., Cai J.L., Yu J.J. (2019). A highly efficient vortex-assisted liquid-liquid microextraction based on natural deep eutectic solvent for the determination of Sudan I in food samples. RSC Adv..

[39] Liu C., Liu D.H., Liu X.K., Jing X., Zong F.L., Wang P., Zhou Z.Q. (2017). Deep eutectic solvent-based liquid phase microextraction for the determination of pharmaceuticals and personal care products in fish oil. New J. Chem..

[40] Wang H.Z., Huang X.D., Qian H., Lu R.H., Zhang S.B., Zhou W.F., Gao H.X., Xu D.H. (2019). Vortex-assisted deep eutectic solvent reversed-phase liquid-liquid microextraction of triazine herbicides in edible vegetable oils. J. Chromatogr. A.

[41] Wu X., Zhang X.X., Yang Y.Q., Liu Y.R., Chen X.N. (2019). Development of a deep eutectic solvent-based matrix solid phase dispersion methodology for the determination of aflatoxins in crops. Food Chem..

[42] Torbati M., Mohebbi A., Farajzadeh M.A., Mogaddam M.R.A. (2018). Simultaneous derivatization and air-assisted liquid-liquid microextraction based on solidification of lighter than water deep eutectic solvent followed by gas chromatography-mass spectrometry: An efficient and rapid method for trace analysis of aromatic amines in aqueous samples. Anal. Chim. Acta.

[43] Funari C.S., Sutton A.T., Carneiro R.L., Fraige K., Cavalheiro A.J., Bolzani V.D., Hilder E.F., Arrua R.D. (2019). Natural deep eutectic solvents and aqueous solutions as an alternative extraction media for propolis. Food Res. Int..

[44] Babaee S., Daneshfar A. (2018). Magnetic deep eutectic solvent-based ultrasound assisted liquid-liquid microextraction for determination of hexanal and heptanal in edible oils followed by gas chromatography-flame ionization detection. Anal. Methods.

[45] Karimi M., Dadfarnia S., Shabani A.M.H., Tamaddon F., Azadi D. (2015). Deep eutectic liquid organic salt as a new solvent for liquid-phase microextraction and its application in ligandless extraction and pre-concentraion of lead and cadmium in edible oils. Talanta.

[46] Huang Y., Feng F., Chen Z.G., Wu T., Wang Z.H. (2018). Green and efficient removal of cadmium from rice flour using natural deep eutectic solvents. Food Chem..

[47] Zounr R.A., Tuzen M., Deligonul N., Khuhawar M.Y. (2018). A highly selective and sensitive ultrasonic assisted dispersive liquid phase microextraction based on deep eutectic solvent for determination of cadmium in food and water samples prior to electro thermal atomic absorption spectrometry. Food Chem..

[48] Borahan T., Unutkan T., Turan N.B., Turak F., Bakirdere S. (2019). Determination of lead in milk samples using vortex assisted deep eutectic solvent based liquid phase microextraction-slotted quartz tube-flame atomic absorption spectrometry system. Food Chem..

[49] Altunay N., Elik A., Gurkan R. (2019). Innovative and practical deep eutectic solvent based vortex assisted microextraction procedure for separation and preconcentration of low levels of arsenic and antimony from sample matrix prior to analysis by hydride generation-atomic absorption spectrometry. Food Chem..

[50] Liu R.L., Yu P., Ge X.L., Bai X.F., Li X.Q., Fu Q. (2017). Establishment of an Aqueous PEG 200-Based Deep Eutectic Solvent Extraction and Enrichment Method for Pumpkin (Cucurbita moschata) Seed Protein. Food Anal. Methods.

[51] Svigelj R., Bortolomeazzi R., Dossi N., Giacomino A., Bontempelli G., Toniolo R. (2017). An effective gluten extraction method exploiting pure choline chloride-based deep eutectic solvents (ChCl-DESs). Food Anal. Methods.

[52] Grudniewska A., de Melo E.M., Chan A., Gnilka R., Boratynski F., Matharu A.S. (2018). Enhanced protein extraction from oilseed cakes using glycerol-choline chloride-deep eutectic solvents: A biorefinery approach. ACS Sustain. Chem. Eng..

[53] Lee J., Jung D., Park K. (2019). Hydrophobic deep eutectic solvents for the extraction of organic and inorganic analytes from aqueous environments. Trac-Trend Anal. Chem..

[54] Zhu S.Q., Zhou J., Jia H.F., Zhang H.X. (2018). Liquid-liquid microextraction of synthetic pigments in beverages using a hydrophobic deep eutectic solvent. Food Chem..

[55] Ravandi M.G., Fat’hi M.R. (2018). Green effervescence assisted dispersive liquid-liquid microextraction based on a hydrophobic deep eutectic solvent for determination of Sunset Yellow and Brilliant Blue FCF in food samples. New J. Chem..

[56] Faraji M. (2019). Determination of some red dyes in food samples using a hydrophobic deep eutectic solvent-based vortex assisted dispersive liquid-liquid microextraction coupled with high performance liquid chromatography. J. Chromatogr. A.

[57] Deng W.W., Yu L., Li X., Chen J., Wang X.X., Deng Z.X., Xiao Y.X. (2019). Hexafluoroisopropanol-based hydrophobic deep eutectic solvents for dispersive liquid-liquid microextraction of pyrethroids in tea beverages and fruit juices. Food Chem..

